# Women with Autoimmune Thyroiditis have Lower Reproductive Life Span or Not? A Cross- Sectional Study

**DOI:** 10.30476/ijcbnm.2020.84255.1207

**Published:** 2020-10

**Authors:** Alamtaj Samsami, Leila Ghasmpour, Shaghayegh Moradi Alamdarloo, Sara Davoodi, Jamshid Rahmati, Ali Karimian, Mona Tavasoli

**Affiliations:** 1 Infertility Research Center, Department of Obstetrics and Gynecology, School of Medicine, Shiraz University of Medical Sciences, Shiraz, Iran; 2 Department of Anesthesiology, School of Medicine, Shiraz University of Medical Sciences, Shiraz, Iran; 3 Department of Physiology, School of Medicine, Shiraz University of Medical Sciences, Shiraz, Iran

**Keywords:** Female infertility, Hashimoto disease, Anti-Müllerian hormone, Ovarian reserve

## Abstract

**Background:**

Autoimmune diseases are a main cause of primary ovarian insufficiency. This study was designed to elucidate the relationship between ovarian reserve and anti-thyroid peroxidase antibodies in women of different ages.

**Methods:**

98 women in a cross-sectional study was conducted at the infertility Center of Shiraz University of Medical Sciences, Hazrate Zeinab Hospital, from September 2018 to March 2019. Women with infertility and thyroid-stimulating hormone (TSH) &gt; 3mIU/L were included in the study with convenience sampling. Data were collected by a form containing demographic characteristics, thyroid hormones, and ovarian reserve data. Participants were categorized based on the negative or positive anti-thyroid peroxidase (anti-TPO) antibodies. According to a pilot sample, and possible dropout of 20%, the sample size was determined to be 49 cases in each group. The results were compared between groups using SPSS, version 22. All statistical comparisons were performed using the t-test, and the data are presented as the mean±SD. P-values less than 0.05 were considered significant.

**Results:**

49 women were analyzed in each group. There was no significant difference between the groups in the mean age (P=0.42), body mass index (BMI) (P=0.34), duration of infertility (P=0.99), mean prolactin (P=0.66), TSH (P=0.17), thyroxine (T4) (P=0.87) and follicle-stimulating hormone (FSH) levels (P=0.14). Ovarian reserve characteristics: antral follicular count (AFC) and anti-Müllerian hormone (AMH) levels in anti-TPO positive group were 10.61±7.29 and 1.98±2.38, respectively, and AFC and AMH in anti- TPO negative group were 16.46±6.38 and 2.94±2.22, respectively. There were significant differences between the two groups on AFC (P <0.001) and AMH (P=0.04).

**Conclusion:**

Patients with autoimmune thyroiditis were at higher risk for decreased ovarian reserve. They should consider their lower reproductive life span during their childbearing years.

## INTRODUCTION

Decreased ovarian reserve is an important cause of infertility among women. ^1^ These women have regular menses but lower responses to ovarian stimulation with fertility treatments, and have lower live birth rates per reproductive cycle. Although ovarian reserve evaluation to identify women who are at risk of decreased ovarian reserve cannot predict the life span of their reproductive years, results outside the normal range may encourage them to become pregnant as soon as possible. At present, anti-Müllerian hormone (AMH) testing is considered the best approach to determine ovarian reserve, given that follicle stimulating hormone (FSH) levels can change during the menstrual cycles. ^2^

Although there are some known causes for primary ovarian insufficiency (POI), the etiology remains unclear in 70–90% of the cases. Several factors are known as the cause of decreased ovarian reserve, e.g. pelvic radiation, previous chemotherapy, smoking, genetic causes such as fragile X mental retardation and permutation, 45X aneuploidy. ^3^ Ovaries are at risk of autoimmune disorders. Some researchers reported high relation in POI and for at least one organ-specific auto-antibody, and the autoimmune disease associated most frequently with POI is autoimmune thyroid disease. ^4^

Hashimoto’s thyroiditis (HT), characterized by infiltrating lymphocytic changes in the thyroid gland, is the most common organ-specific autoimmune disease of thyroid, affecting 18% of the population. ^5^ The European Society of Human Reproduction and Embryology consensus of 2017 suggested thyroid stimulating hormone (TSH) and anti thyroid peroxidase (anti-TPO) evaluation for women with POI who have unknown cause. ^6^ The study on 5000 women showed no ovarian reserve impairment in patients with HT. ^7^ The study by Tuten et al. presented higher AMH in these patients. ^8^ However, the other paper showed a decrease in the AMH level in patients with autoimmune thyroiditis. ^9^ In patients with autoimmune thyroiditis, prescription of levothyroxine helps improve follicular status and increasing AMH level. ^10^ Incidence rates of 2-7.6 per 1000 person in overt and subclinical hypothyroidism in Tehran thyroid study suggest that we should consider all aspects of health in these patients. ^11^

Some papers show a relationship between AMH and immune problem of autoimmune thyroiditis patients. ^1 , 9^ Finding a relationship between decreased ovarian reserves and autoimmune thyroiditis is an important key in saving fertility in this group of women. The aim of this study was to investigate the relationship between autoimmune thyroiditis and ovarian reserve criteria based on women’s age. 

## METHODS

This cross-sectional study was conducted at the infertility Center of Shiraz University of Medical Sciences, Hazrate Zeinab Hospital, from September 2018 to March 2019. In this time period, all infertile patients who met the inclusion criteria were included in the study using convenience sampling. Data were collected by a form containing demographic data, body mass index, TSH, thyroxine (T_4_), anti-TPO antibody, prolactin, AMH, FSH, luteinizing hormone (LH), and antral follicular count (AFC). Thyroid aut- antibody levels were measured as anti-TPO with a Cobas e411 analyzer, and values higher than 25 IU/mL were considered positive for HT.

The inclusion criteria were: women who had informed consent, TSH level higher than 3 mIU/L, normal serum prolactin, age between 18 and 42 years, and primary or secondary infertility (failure to achieve clinical pregnancy after 1 year of regular unprotected intercourse. ^12^ The exclusion criteria were BMI>35 kg/m^2^, and any other autoimmune disease. All participants meeting the inclusion criteria were divided into two groups based on the positive or negative anti-TPO levels. According to a pilot sample, for comparing AMH between positive and negative Anti-TPO groups, the mean and standard deviation of these two groups were 3.04±1.68 and 1.96±1.75, respectively.

Then, considering β=0.2, α=0.05, S_1_=1.68, S_2_=1.75, μ_1_=3.04, and μ_2_=1.96, 41 subjects were estimated to be needed in each group. However, given a possible dropout of 20% (n'=n×$\frac{1}{\mathrm{1-p}}$), the sample size was determined 49 cases in each group.

$n=\frac{(S_{1}^{2}+S_{2}^{2}){\left(Z_{1-\frac{\alpha }{2}}+Z_{\mathrm{1-\beta}}\right)}^{2}}{d^{2}}=41$

d=μ_1_-μ_2_=1.08 ,α=0.05,1-β=0.8

$Z_{1-\frac{\alpha }{2}}=1.96$ , $Z_{\mathrm{1-\beta}}=0.84$ , $S_{1}^{2}+S_{2}^{2}=5.88$

The medical history and findings on physical examination were recorded for each woman, and then ovarian function was determined as baseline FSH on the second or third days of the menstrual cycle, and AMH, TSH and prolactin concentrations were also measured by 5cc blood which were taken after informed consent was obtained. AFC was evaluated in all women by transvaginal ultrasonography on the second or third days of their menstrual cycle.

The effect of autoimmune thyroiditis on ovarian reserve was determined as differences in AMH, AFC and FSH levels between the groups. The anti-TPO positive and -negative groups were further subdivided into 2 age groups to investigate the possible effect of age. The results were compared between groups using SPSS software, version 22. All statistical comparisons were performed using the t-test, and the data are presented as the mean±SD. P-values lower than 0.05 were considered significant.

A written informed consent was obtained from all participants. The study protocol was approved by the ethics committee of the Shiraz University of Medical Sciences (IR.sums.med.rec.1397.318)

## RESULTS

A total of 98 women with infertility were included, and the results for 49 patients in each group were used in the analysis. The patients’ mean age (P=0.42), BMI (P=0.34), duration of infertility (P=0.99), mean prolactin (P=0.66), TSH (P=0.17), thyroxine (P=0.87) or FSH level (P=0.14) did not differ significantly between the groups, as shown in Table 1.

**Table 1:** Clinical characteristics of the study groups

Variable	Anti-TPO^a^ positive group (N=49)	Anti-TPO negative group (N=49)	P value*
	mean±SD	mean±SD	
Age (year)	32.22±5.40	32.40±4.69	0.42
BMI^b^ (kg/m^2^)	26.90±3.83	26.22±3.25	0.34
Duration of infertility (years)	7.38±4.29	7.39±4.79	0.99
Prolactin (μg/L)	14.64±7.53	15.30±7.50	0.66
TSH^c^ (mIU/L)	5.57±4.36	4.65±1.64	0.17
T_4_^d^ (μg/dL)	8.98±4.14	9.11±4.07	0.87

* t test;

a Anti-thyroid peroxidase;

b Body mass index;

c Thyroid-stimulating hormone;

d Thyroxine

 Ovarian reserve was compared between the groups with negative and positive anti-TPO findings in Table 2. Ovarian reserve characteristics: AFC and AMH levels in anti-TPO positive group were 10.61±7.29 and 1.98±2.38, respectively, and AFC and AMH in anti- TPO negative group were 16.46±6.38 and 2.94±2.22, respectively. There were significant differences between the two groups on AFC (P<0.001) and AMH (P=0.04), indicating significantly lower mean AFC and AMH levels in the anti-TPO positive group. Age-related differences in ovarian reserve in each group are shown in Table 3 for women younger than 35 years of age, and in Table 4 for women 35 years old and older. In women younger than 35 years, only mean AFC level was lower, but in the older age group both AMH and AFC levels were lower in the anti-TPO positive group.

**Table 2:** Ovarian reserve characteristics of the study groups

Variable	Anti-TPO^a^ positive group (N=49)	Anti-TPO negative group (N=49)	P value*
	mean±SD	mean±SD	
FSH^b^ (mIU/mL)	7.52±6.34	6.08±2.59	0.14
AMH^c^ (ng/mL)	1.98±2.38	2.94±2.22	0.04
AFC^d^ (number)	10.61±7.29	16.46±6.38	<0.001

* t test;

a Anti-thyroid peroxidase;

b Follicle-stimulating hormone;

c Anti-Müllerian hormone;

d Antral follicular count

**Table 3:** Age-related ovarian reserve characteristics in women younger than 35 years

Variable	Anti-TPO^a^ positive group (N=27)	Anti-TPO negative group (N=34)	P value*
	mean±SD	mean±SD	
AMH^b^ (ng/mL)	2.36±2.85	3.00±2.35	0.39
FSH^c^ (mIU/mL)	7.10±6.98	6.06±3.04	0.44
AFC^d^ (number)	12.14±7.13	17.38±6.01	<0.001

* t test;

a Anti-thyroid peroxidase;

b Anti-Mllerian hormone;

c Follicle-stimulating hormone;

d Antral follicular count

**Table 4:** Age-related ovarian reserve characteristics in women aged 35-year-old or older

Variable	Anti-TPO^a^ positive group (N=22)	Anti-TPO negative group (N=15)	P value*
	mean±SD	mean±SD	
AMH^b^ (ng/mL)	1.51±1.58	2.81±1.98	0.03
FSH^c^ (mIU/mL)	6.75±5.72	6.12±1.15	0.68
AFC^d^ (number)	8.72±7.19	14.40±6.93	0.02

* t test;

a Anti-thyroid peroxidase;

b Anti-Mllerian hormone;

c Follicle-stimulating hormone;

d Antral follicular count

## DISCUSSION

This study revealed that anti-TPO positive women had AMH level and AFC significantly lower than the anti-TPO negative women. The other predictor of ovarian reserve, FSH, was higher in the anti-TPO positive group, but the difference between the groups was not significant. FSH and LH in other studies have been evaluated in poly-cystic ovary syndrome and higher ratio of LH to FSH, ^13^ but in the literature there was a controversy about these laboratory data in POI patients. Regarding the association between the AMH level and age, there was no significant difference between the groups in women younger than 35 years, but in women aged 35 years and older, levels of this hormone were significantly lower in the anti-TPO positive group in this study. This was confirmed in the studies done in lower means of age. ^14 - 16^ Likewise, AFC – another criterion for ovarian reserve – was significantly lower in anti-TPO positive women in both age groups. This difference may have occurred because AFC is an operator-dependent criterion, whereas AMH is a more reliable predictor of ovarian reserve ^17^

The difference between the groups in the FSH level was not significant, which might be because the women in our sample had not reached menopause and they all had regular menstrual cycles. Two studies published in 2016 and 2018 on ovarian reserve in adolescent girls showed that serum AMH levels were not affected by HT. ^14 , 15^ Autoimmune damage to the ovaries may take longer to become detectable, and the teenage years might be too early to see these effects. This study in reproductive age and advanced ages in comparison with teenagers presented different results. Follow-up of patients for reproductive abnormalities and prospective studies is needed to confirm this possibility.

A recent longitudinal study showed that women with low ovarian reserve had higher baseline levels of TPO antibody, and that these levels increased during a12-year follow-up period. These authors also found increasing levels of anti-TPO antibody with time. Comprehensive prospective studies with periodic determinations of AMH level and thyroid function test make it important to find a relationship between ovarian reserve and thyroid function. ^16^ Although the final conclusion of this study was consistent with the present study, aformentioned study had a strength point of following up the autoimmune thyroiditis patients.

One previous study found that the prevalence of primary infertility in Iran was higher than the global infertility rate, and that ovulatory problems were the main cause. Knowledge of the risk factors for infertility is important to enable women to make decisions on the timing of conception, and is also important for healthcare providers and policy makers responsible for designing and providing interventions aiming at reducing the downward trend in fertility. ^18^

A researcher reported a study with about 5000 infertile women and presented positive anti-TPO in 9.8%-12.1% that is higher in low ovarian reserve patients. They showed more thyroid disorder in infertile women. ^7^ The present study showed that ovarian reserve was lower in a sample of women in Iran with autoimmune thyroiditis. We suggest that women with autoimmune thyroiditis should be informed about its effects on their ovarian reserve and fertility, and should be counseled about the implications for their family planning. In light of the present findings, women with decreased ovarian reserve should be advised to plan for conceiving earlier in their reproductive life span. Since their opportunity for pregnancy may be lower than what is predicted.

The strength of this study is evaluation of anti- TPO positive test in women with complaint of infertility. The limitation was the small sample size, which was restricted in the anti-TPO positive women with infertility complaint.

## CONCLUSION

In conclusion, women with autoimmune thyroiditis might be at higher risk of ovarian insufficiency because of the significant decrease in ovarian reserve. Patients with positive anti-TPO antibody findings who have not yet completed their family should be aware of their lower reproductive life span before seeking medical consult for infertility. This paper recommends long term prospective studies for follow up in autoimmune disease, especially in young women and juvenile’s reproductive system. More studies in women without complaint of infertility will help finding the best screening marker in primary ovarian insufficiency.
